# Quantitative Measurements of DP in Cellulose Paper Based on Terahertz Spectroscopy

**DOI:** 10.3390/polym15010247

**Published:** 2023-01-03

**Authors:** Qiyu Chen, Lijun Yang, Hua Yu, Yuxin He, Hong Liu, Xuan Wang

**Affiliations:** 1 State Key Laboratory of Power Transmission Equipment & System Security and New Technology, Chongqing 400044, China; 2 State Grid Shanxi Electric Power Research Institute, Taiyuan 030001, China

**Keywords:** insulating paper, cellulose, degree of polymerization (DP), FTIR spectra, molecular simulation, terahertz spectroscopy, non-destructive detection

## Abstract

The power transformer is vital to the reliability of the power grid which is most commonly insulated with Kraft paper and immersed in mineral oil, among which the aged state of the paper is mainly correlated to the operating life of the transformer. Degree of polymerization (DP) is a direct parameter to assess the aged condition of insulating paper, but existing DP measurement by viscosity methods are destructive and complicated. In this paper, terahertz time-domain spectroscopy (THz-TDS) was introduced to reach rapid, non-destructive detection of the DP of insulating paper. The absorption spectra of insulating paper show that characteristic peak regions at 1.8 and 2.23 THz both exhibit a log-linear quantitative relationship with DP, and their universalities are confirmed by conducting the above relationship on different types of insulating paper. Fourier transform infrared spectroscopy (FTIR) analysis and molecular dynamics modeling further revealed that 1.8 and 2.23 THz were favorably associated with the growth of water–cellulose hydrogen bond strength and amorphous cellulose, respectively. This paper demonstrates the viability of applying THz-TDS to the non-destructive detection of DP in insulating paper and assigned the vibration modes of the characteristic absorption peaks.

## 1. Introduction

The service life of oil-immersed power transformers is typically determined by the condition of the oil-paper insulating [1,2,3], and the degree of polymerization (DP) of the insulating paper is the most direct and reliable indicator of the oil-paper insulation condition [4]. Currently, the DP value is measured mainly regarding the viscosity method outlined in IEC 60450 [5], which is vulnerable to test procedures and destructive to the samples [6,7]. In the last few decades, finding a fast, non-destructive method to value the DP of insulating paper has become one of the most important goals of researchers [8,9].

Near-infrared spectroscopy (NIR) is the most representative method to reflect the aged condition of insulating [10,11,12,13]. At the beginning of the 21st century, the research of the G.C. Stevens group at Surrey University, U.K. proposed to establish NIR databases of insulating paper samples that were differently degraded and combined multivariate statistical methods to predict the DP of unknown samples [14,15]. Additionally, Huazhong University of Science and Technology [16] and Xi’an Jiaotong University [17] conducted research to improve the DP prediction accuracy of unknown samples by increasing the number of specimens and optimizing the fitting algorithm. At present, NIR has not been applied to the engineering application in non-destructive testing of DP. There are two main reasons for limiting its development. First, the NIR describes the vibration of the functional groups in the molecule, however, the decrease in DP caused by thermal aging has little effect on the functional group types of cellulose, making it difficult to identify the characteristic peaks associated with DP in the NIR spectrum. Currently, most of them rely on stoichiometric methods and statistical models to predict the DP of insulating paper [18]. The accuracy of the prediction is heavily dependent on the sample database and also susceptible to environmental factors [19]. Second, the insulating oil as well as the color of the oil, affect the absorbance of the full band, which makes modeling extremely difficult [20]. The NIR method cannot meet the engineering requirements in DP testing, therefore researchers continue to seek out new non-destructive testing technologies in recent years.

In the thermal aging process of insulating paper, the decrease in DP is accompanied by the change of hydrogen bond (H-bond) structure. Terahertz technology has been gradually noticed in the field of non-destructive testing of insulating material because of its unique response of H-bond structure change and high transmittance to insulating oil terahertz radiation (T-Ray), which refers to electromagnetic radiation between infrared radiation and microwave over the frequency range of 0.1 to 10 THz and a wavelength range of 30 μm to 3 mm. Terahertz time-domain spectroscopy (THz-TDS) has a sensitive response to the H-bond structure of cellulose [21,22,23,24,25,26]. The decrease in DP of insulating paper leads to changes in the structure and strength of the H-bond network [27]. Theoretically, during the thermal aging process, the THz spectra of insulating paper are sensitive to the changes in the cellulose H-bond network, which indicates that DP-related information may be extracted from the spectra [28,29,30,31,32,33]. In addition, compared with the NIR method, THz spectroscopy is highly penetrating for weakly polar substances such as insulating oil, and the state of the insulating oil has less influence on the test results, facilitating a higher signal-to-noise ratio to be obtained.

The current researchers’ experience in identifying the conformational changes of H-bond network in cellulose through THz-TDS has laid a solid foundation for this article. In 2012, Kurabayashi et al. discovered that THz waves are sensitive to the structural variations of cellulose and may be utilized to distinguish between natural and synthetic fibers [28]. In 2014, Guo Hong et al. [34] demonstrated through experiments that THz spectroscopy has the potential to accurately assess the strength of H-bonds in cellulose compared with those obtained from X-ray diffraction (XRD) and Fourier transform infrared spectroscopy (FTIR) analysis. In 2017, a correlation was found between the THz absorption peaks of fibers and the variation of H-bond network and crystalline regions and applying this feature to detect the aged condition of ancient paper and fiber products was proposed. In 2018 [35], Italian scholar M. Missouri also conducted a study on the polymerization degree and THz spectral absorption coefficients of ancient paper and quantified the relationship [36]. Therefore, the advantages of THz waves including a unique response to molecular low-energy vibrations and good penetrability to weakly polar substances [37], make THz-TDS promising for overcoming the limitations of conventional spectroscopic methods and pave the way for rapid, non-destructive detection of the DP.

In addition to cellulose, the effects of substances such as lignin and hemicellulose on the terahertz spectroscopy should be considered. Insulating paper is composed of around 90% cellulose, and 6–7% lignin, and the remainder is hemicellulose (a polymer of pentose and hexose) [38,39]. It is generally accepted that lignin causes higher absorption than cellulose [1], and that lignin will influence the degradation of cellulose [40,41,42,43]. Due to its low thermal stability [44], hemicellulose may be degraded at the hotspots of insulation in the transformer. While compared to cellulose, the absorption coefficient of lignin and hemicellulose have no order-of-magnitude difference in the range of interest here, i.e., <3 THz [45,46]. As a weakly polar substance, insulating oil absorbs small amounts of terahertz waves [47]. To simplify the analysis, this paper established a preliminary DP evaluation model not considering the effect of the lignin, hemicellulose, and insulating oil, which is also a part of the model error. Future studies on the terahertz absorption of lignin and other compounds can further optimize the model in Section 3.2. In this paper, the THz-TDS technique is utilized to reach the quantitative measurements of DP in insulating paper. In Section 2, we prepared degraded insulating paper with different DP values through thermal aging experiments and described the methods of acquiring terahertz, infrared spectrum, and data processing. In Section 3, the absorption spectra were calculated, and the characteristic peaks and parameters related to the change of DP were extracted by baseline correction and peak fitting algorithm. In Section 4, combined with FTIR and molecular dynamics simulation, the vibration modes of characteristic peaks associated with DP changes are explained.

## 2. Material and Methods

### 2.1. Sample Preparation and Experiments

**Figure 1:**
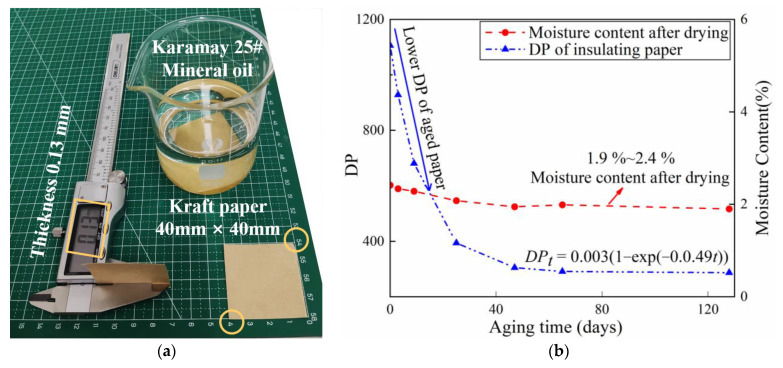
(**a**) The oil-paper insulation materials. (**b**) DP and moisture content of the insulating paper.

Oil-paper insulating samples were prepared using 25# Karamay naphthenic mineral oil (PetroChina Co., Ltd., Karamay, China) and 0.13 mm-thick ordinary kraft insulating paper (Weidmann Power Insulation Technology Co., Ltd., Taizhou, China) with an initial DP of 1100 as shown in Figure 1a. Each paper sample was cut to a size of 40 mm × 40 mm. The oil and paper were dried in a 50 Pa vacuum at 90 °C for 48 h, respectively. Several samples of insulating oil and paper were prepared following an oil–paper ratio of 10:1 and injected into the container. Then, the insulating oil and paper samples were mixed at 90 °C for 72 h in a 50 Pa vacuum to remove moisture and gas further. After the oil impregnation, the samples were sealed and placed in an aging chamber at 130 °C for 0, 3, 9, 19, 25, 37, 47, 65, and 128 days.

The DP and moisture content after drying of aged paper were evaluated. The DP was determined using the viscosity method following IEC 60450 [48], and the moisture content was determined using the Karl Fischer titration method following IEC 60814 [49]. In the determination procedure of DP, hexane was used to wash the paper samples at least five times to remove the insulating oil and eliminate its influence on the viscosity test. The aged paper samples were dried at 90 °C for 4 h and then stored in a dry insulating oil bottle to maintain a constant moisture content before collecting the terahertz spectra. Figure 1b depicts the variations in DP value and moisture content as insulating paper ages. DP and moisture content of insulating paper decreased as thermal aging days increased, with DP decreasing from 1106 to 287 and moisture content decreasing from 2.4% to 1.6%. Figure 1b shows that the moisture content of the dried insulation paper is positively in proportion to the DP value. During the thermal aging process, while the DP decreases the hydrophilic groups on the cellulose molecular chain are destroyed, resulting in a decrease in water-absorbing ability. Therefore, after drying, the moisture content of the aged insulation paper is lower. 

### 2.2. THz Spectroscopy Acquisitions

**Figure 2:**
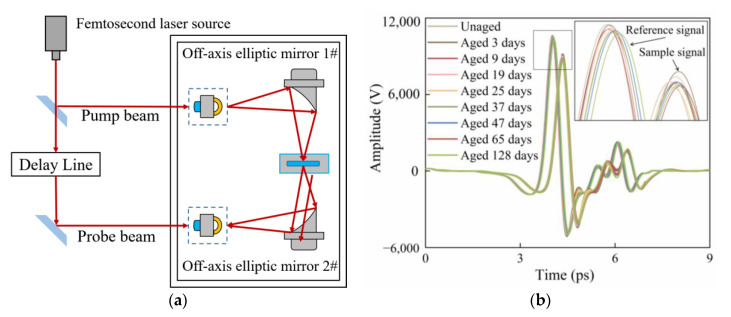
(**a**) Schematic diagram of transmission THz-TDS system. (**b**) Terahertz time-domain spectra of air reference and insulating paper with different aging days.

Figure 2a depicts the schematic diagram of the transmission THz-TDS system. The femtosecond laser in the spectrometer emits a femtosecond laser pulse, which is split by a beam splitter into the pump beam and probe beam. The pump beam is focused onto the emitter’s surface by off-axis elliptic mirror 1# to generate THz pulses. After the THz pulses transmit through the sample, the THz pulses carrying sample information are collimated and focused by off-axis elliptic mirror 2# and pass through the THz detector collinearly with the probe beam through a delay line. The signal is finally sent to the computer for further analysis.

The reference and sample spectrums can be acquired using the system described above. THz signals were recorded three times at different points on each sample and then averaged for each sample. The temperature and relative humidity of the air was maintained at 25 °C and 20%, respectively. Figure 2b exhibits the terahertz time-domain spectra of reference and sample.

### 2.3. THz Data Processing

The Dorney [50] and Duvillaret [51] mathematical model was used to calculate the absorption coefficient of the insulating paper sample over the range of 0.2–2.5 THz. 

Fast Fourier transform was performed on the terahertz time-domain spectra of air and $E_{\mathrm{re}f}(t)$ samples $E_{sam}(t)$ to obtain the frequency spectra $E_{ref}(\omega )$ and *E_xample_*(*ω*). The complex transmission function *T*(*ω*) of the sample is expressed as in Equation (1):

(1) $$T(\omega )=\frac{E_{ref}(\omega )}{E_{sam}(\omega )}=\frac{4\tilde{n}(\omega )}{{\left(\tilde{n}(\omega )+1\right)}^{2}}e^{i2\pi (\tilde{n}(\omega )-1)d\omega /c}=\rho (\omega )e^{\varphi (\omega )}$$

The complex refractive index $\tilde{n}(\omega )=n(\omega )-j\kappa (\omega )$ is substituted into Equation (1), where κ(ω) is the extinction coefficient of the sample, which describes the absorption degree of the medium to the THz wave. Therefore, a conversion relationship exists between κ(ω) and the absorption coefficient α(ω), as α(ω) may be further deduced from the following equations:

(2) $$n(\omega )=\varphi (\omega )\frac{c}{\omega d}+1=1+\frac{c}{\omega d}\left[\varphi_{s}(\omega )-\varphi_{ref}(\omega )\right]$$

(3) $$\kappa (\omega )=\frac{c}{\omega d}\ln\left[\frac{4n(\omega )}{\rho (\omega ){\left[n(\omega )+1\right]}^{2}}\right]=\frac{c}{\omega d}\ln\frac{\left|E_{ref}(\omega )\right|}{\left|E_{s}(\omega )\right|}$$

(4) $$\alpha (\omega )=\frac{2\kappa (\omega )\omega }{c}=\frac{2}{d}\ln\left[\frac{4n(\omega )}{\rho (\omega ){\left[n(\omega )+1\right]}^{2}}\right]=\frac{2}{d}\ln\frac{\left|E_{ref}(\omega )\right|}{\left|E_{s}(\omega )\right|}$$

where c is the speed of light in vacuum, *ω* is the angular frequency, *d* is the sample thickness, ρ(ω) and φ(ω) is the amplitude ratio and phase difference of reference and sample signal, respectively.

### 2.4. Molecular Structure from FTIR Analysis

The FTIR spectra of aged insulating paper were collected to analyze the molecular structure changes during the thermal aging process. The Nicolet iS5 FTIR spectrometer (Thermo Fisher Scientific, Waltham, MA, USA) was used and the spectral acquisition was obtained through the attenuated total reflectance detector. For each spectrum, a total of 16 scans with a 4 cm^−1^ resolution were collected over the range of 4000~400 cm^−1^. The background air was scanned before each measurement.

## 3. THz Spectroscopy Analysis

### 3.1. THz Absorption Spectra Analysis

**Figure 3:**
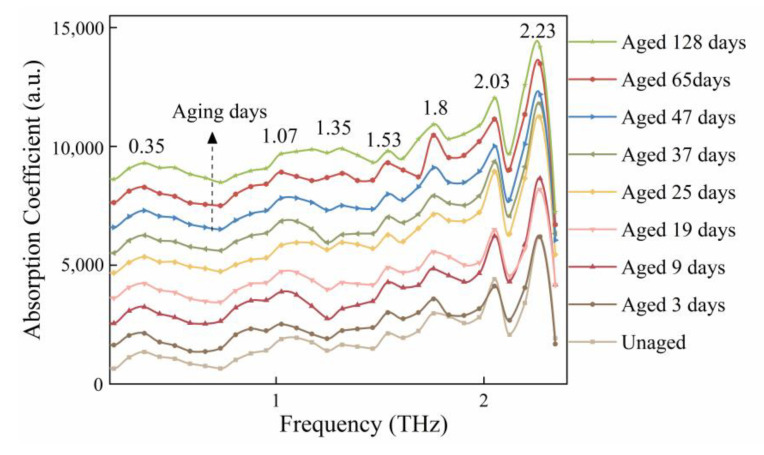
Absorption spectra of the insulating paper with different aging days.

Figure 3 depicts the terahertz absorption spectra of insulating paper samples after various aging intervals. For ease of observation, these spectra were moved by the same distance along the vertical coordinate. Multiple absorption peaks can be seen in the spectra ranging from 0.2 to 2.5 THz for each sample, particularly at 1.80, 2.03, and 2.23 THz.

### 3.2. Baseline Correction and Parameters Extraction

First, the baseline of the absorption spectra is corrected to minimize the effects of background noise, pulsed light source instability, and humidity fluctuations. Among the algorithms for automatic baseline deduction in spectra, the adaptive iterative reweighting penalized least squares (airPLS) is a method with high accuracy. AirPLS is a baseline fitting method that does not require the identification of the peak region [52]. It employs the iterative adaptive method to obtain the weight vector *w*, assigns weights by function, and imposes a penalty to control the baseline’s stability. Assuming that the length of the signal ***y*** is *N* and the initial value of the baseline weights is 1, once the first solution of the baseline ***z*** is determined, the baseline weights are iteratively updated to produce an updated baseline. If *y_i_* ≥ *z_i_*, the weight coefficient *w_i_* of the signal is set to 0; otherwise, the weight is updated according to Equation (5). The iteration terminates either when the maximum number of times is reached or when |d|<0.001×|y| is satisfied.

(5) $$w_{i}=\left\{\begin{array}{ll} 0, & y_{i}\geq z_{i}\\ e^{t\left(y_{i}-z_{i}\right)/|d|}, & y_{i}<z_{i} \end{array}\right.,i=1,2,...,N$$

where *y* is the sample signal, *z* is the fitting baseline, *t* is the iteration times, and **d** is the vector that consists of negative elements of the subtraction, *y* − *z*.

**Figure 4:**
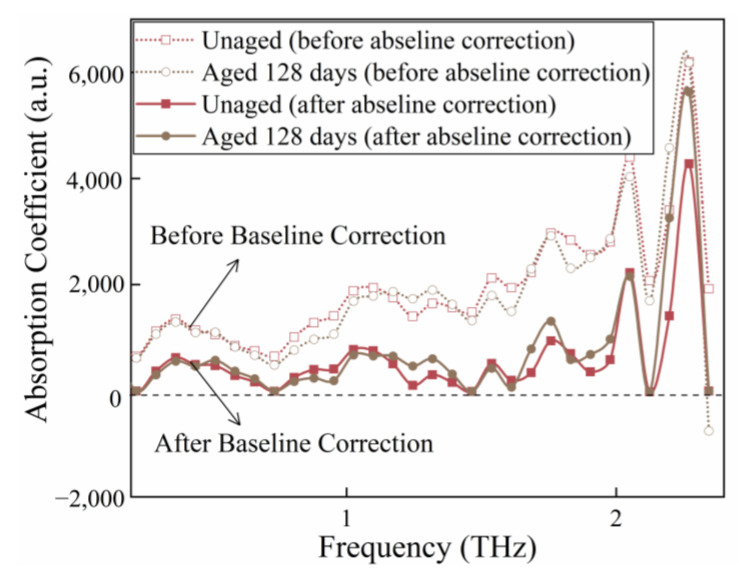
Absorption spectra of the unaged and aged 128 days insulating paper before and after baseline correction.

Figure 4 depicts the absorption spectra before and after baseline correction, where the dotted line represents the original signal, and the solid line represents the corrected signal. As an illustration, the measured terahertz time-domain spectra of unaged and aged 128 days insulating paper are depicted in Figure 4 after baseline correction.

A peak-seeking fitting algorithm was employed to locate the peaks and extract the characteristic peaks correlated with DP values. After debugging the parameters, 15% of the maximum value of the absorption coefficient between 0.2 and 2.5 THz was chosen as the peak-search threshold; the second-order derivative approach was adopted to locate the absorption peaks of the spectra, and the Gaussian function of the Equation (6) was used to fit the spectral curves.

(6) $$g(\omega )=(k/\sqrt{2\pi }\sigma )\exp[-{\left(\omega -\mu \right)}^{2}/(2\sigma^{2})]=A\exp[-{\left(\omega -\mu \right)}^{2}/(2\sigma^{2})]$$

where *μ*, *A*, and *σ* are the central frequency, peak height, and half-peak height and width parameters, respectively; *k* is the proportionality constant; *ω* is the frequency.

**Figure 5:**
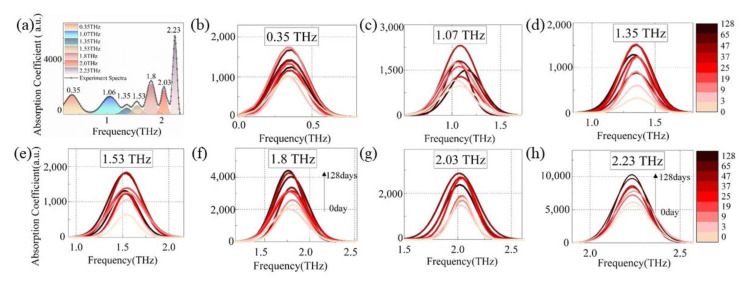
(**a**) Schematic diagram of the multi-peak fitting of the spectrum after baseline correction and the separated absorption peak curves at (**b**) 0.35 THz, (**c**) 1.07 THz, (**d**) 1.35 THz, (**e**) 1.53 THz, (**f**) 1.8 THz, (**g**) 2.03 THz, and (**h**) 2.23 THz.

Taking the absorption spectrum of the unaged insulating paper after deducting the baseline as an example, Figure 5a shows a schematic diagram of the absorption peak localization and multi-peak fitting of the test spectrum, and seven characteristic peaks were found. Using the same split-peak fitting algorithm, Figure 5b–h shows the various patterns of the characteristic peaks of the insulating paper samples aged from 0 to 128 days.

**Figure 6:**
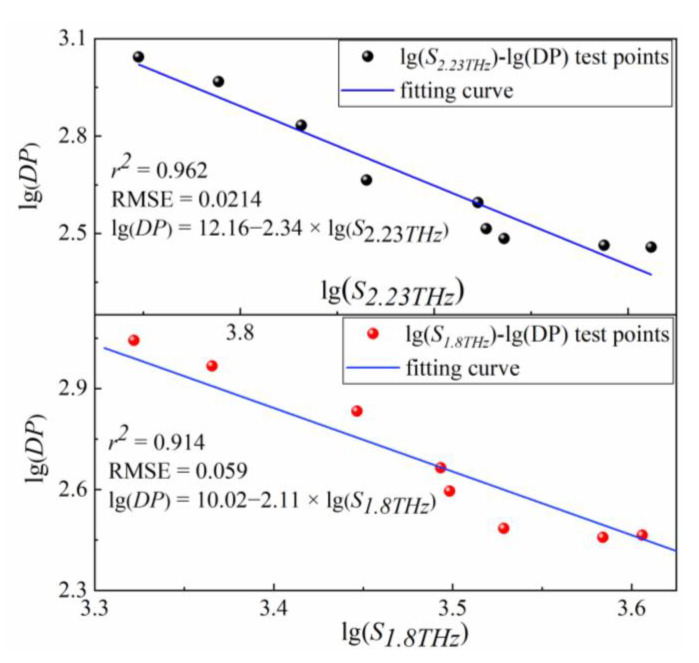
Double-logarithmic linear relationship of lg(*S_1.8THz_*), lg(*S_2.23THz_*), and lg(DP).

Figure 5b–h demonstrates that although the thermal aging process has a significant impact on the intensities of all seven peaks, only two absorption peaks at 1.8 THz and 2.23 THz exhibit a strong positive correlation with DP value. To further quantify this correlation, the peak areas *S_1.8THz_* and *S_2.23TH__z_* were extracted as characteristic parameters and fit with DP values. Figure 6 shows the log-linear fitting curves and regression evaluation indexes. The correlation indices (R^2^) are 0.914 and 0.962, and the root-mean-square errors of prediction (RMSE) are 0.059 and 0.021, respectively, for fitting curves of *S_1.8THz_* and *S_2.23THz_* with DP. In the latter part, *S_1.8THz_* and *S_2.23THz_* were used as the characteristic parameters to quantitatively assess the DP of insulating paper by Equations (7) and (8)

lg(DP) = 10.02 − 2.11 × lg(*S_1.8THz_*) (7)

lg(DP) = 2.812 − 0.05 × lg(*S_2.23THz_*) (8)

### 3.3. Universality Validation of Characteristic Parameters

**Figure 7:**
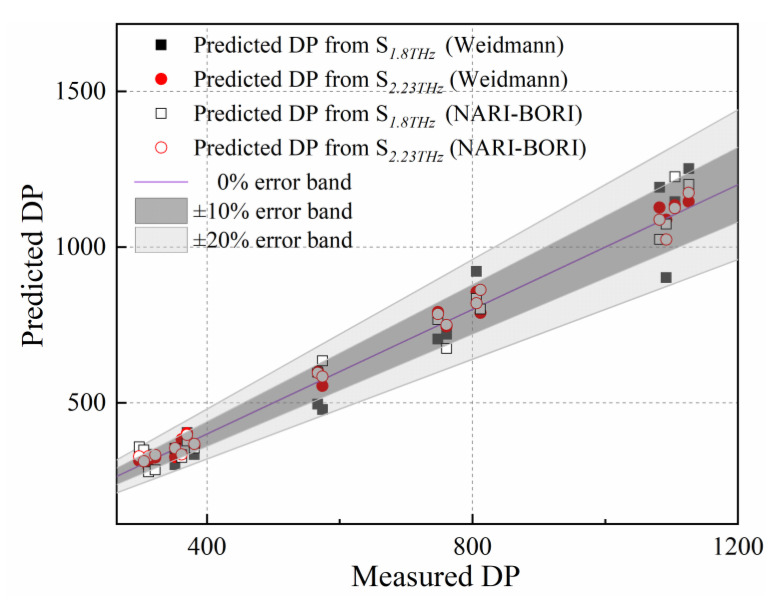
Predicted DP and error band of evaluation model applied in two types of insulating paper obtained from the Weidmann and NARI-BORI companies.

To further examine the universality of characteristic parameters, two batches of 0.13 mm-thick kraft insulating paper purchased from Weidmann (Weidmann Power Insulating Technology Co., Ltd., Jiaxing, China) and NARI-BORI (Chongqing NARI-BORI Transformer Co., Ltd., Chongqing, China) were selected as the sample materials, then thermal aging experiments were reconducted at 130 °C lasting 0, 3, 9, 17, 25, 37, 47, 65, and 128 days. A total of 36 samples were collected as the validation sample set with two samples at each aging day for each type of insulating paper. After baseline correcting and split-peak fitting, the parameters *S_1.8THz_* and *S_2.23THz_* were extracted and substituted into Equations (7) and (8). As shown in the Figure 7, the prediction error bands were 1.4–19.1% and 1.3–9.9% for *S_1.8THz_* and *S_2.23THz_*, respectively, covering two types of insulating paper (purchased from Weidmann and NARI-BORI company), with the prediction error for the *S_2.23THz_* being less.

In conclusion, the parameters to determine the DP of insulating paper based on *S_1.8THz_* and *S_2.23THz_* can be applied in the non-destructive detection of aging state.

## 4. Explanation of THz Vibration Modes 

To explain the correlation between the spectral absorption properties at 1.8 and 2.23 THz and the DP of insulating paper further, theoretical calculations and FTIR analysis of cellulose structure were performed to analyze the spectrum vibration.

### 4.1. Molecular Structure from FTIR Analysis

**Figure 8:**
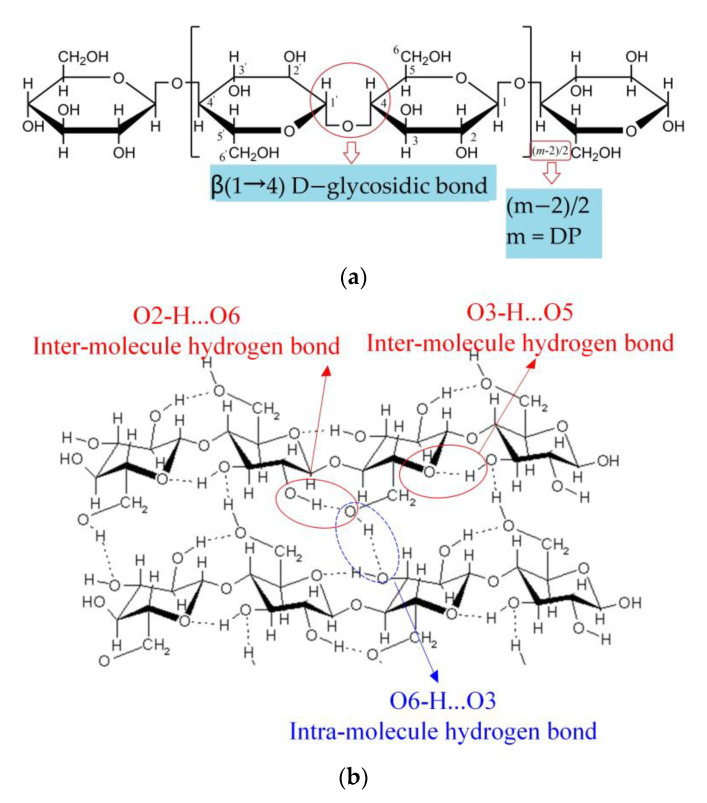
(**a**) Repetitive unit of cellulose. (**b**) Inter- and intra-molecule hydrogen bond structure of cellulose.

As illustrated in Figure 8a, the major component of insulating paper is α-cellulose, where m is the DP of cellulose. As seen in Figure 8, cellulose has a repeat unit of cellobiose linked by β (1→4) D-glyosidic bond, and cellulose chains are linked by inter-molecular and intra-molecular H-bonds. The thermal aging of cellulose is characterized primarily by changes in molecular structure and water molecule adsorption behavior. Changes in molecular structure are mostly expressed in the transition from crystalline to amorphous regions, where density declines and disorder rises. In addition, when cellulose ages, water molecules are consumed, generated, and then moved to various positions in cellulose chains to form various kinds of water–water and water–cellulose H-bonds. Consequently, this paper uses infrared spectroscopy to analyze the H-bond structure and molecular crystal phase changes of insulating paper.

**Figure 9:**
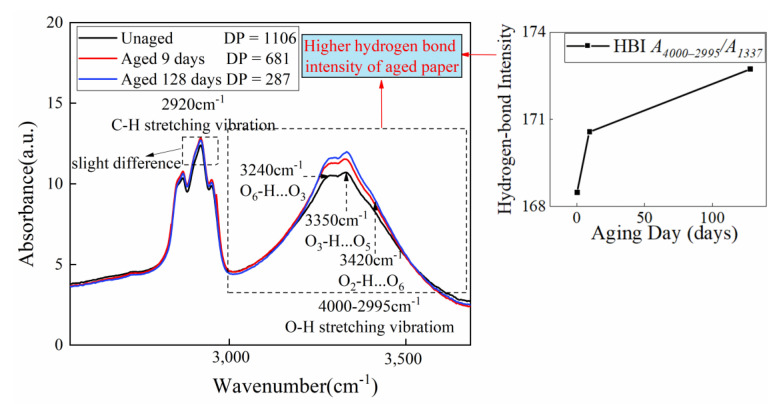
FTIR spectra of insulating paper aged 0, 9, and 128 days including the C-H and O-H stretching vibration bands.

Figure 9 shows the FTIR spectra of the insulating paper before and after the thermal aging experiment over a wavenumber range of 4000–2995 cm^−1^, which is the superposition of multiple -OH stretching vibration bands. In the 4000–2995 cm^−1^ band, 3600–3200 cm^−1^ is assigned to the O-H stretching vibration, while 3455–3410, 3375–3340, and 3310–3230 cm^−i^ are attributed to the intramolecular O_2_–H…O_6_, O_3_–H…O_5_, and intermolecular O_6_–H…O_3_ H-bonds (seen in Figure 8b), respectively. Therefore, the ratio of the absorbance at 4000–2995 cm^−1^ to those at 1337 cm^−1^ due to C-OH in-plane stretching can be used to quantify hydrogen-bond intensity(HBI) [53] as depicted in Figure 9, indicating that the H-bond structure’s strength of aged insulating paper increased. The pores between cellulose chains become larger due to the thermal aging process, making it easier for water molecules to bind to cellulose and increasing the amount of water–cellulose hydrogen bonds, which results in a rise in HBI.

**Figure 10:**
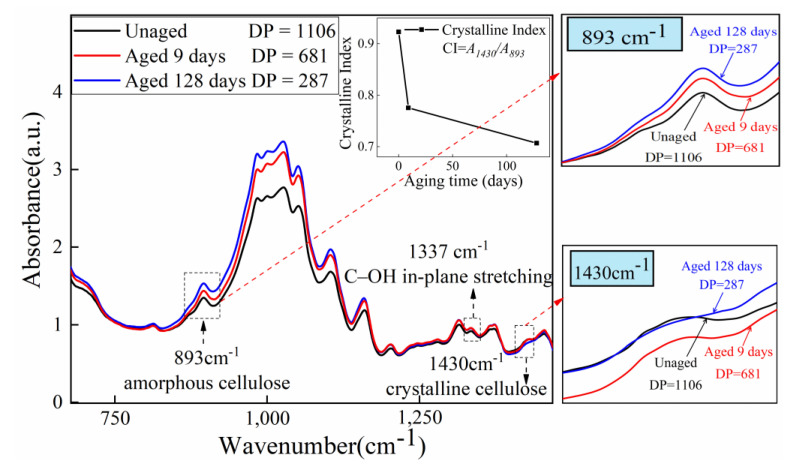
FTIR spectra of insulating paper aged 0, 9, and 128 days including the 893, 1337, and 1430 cm^−1^ bands.

Further analyzing the crystal structure of cellulose, the bands at 893 cm^−1^ (attributed to vibration of β-glyosidic bond and C_1_-H) and 1430 cm^−1^ (attributed to stretching vibration of CH_2_) are sensitive to the amorphous region and crystalline region, respectively. For cellulose to experience the thermal aging process, the peaks at 1430 cm^−1^ become weaker while the peaks at 893 cm^−1^ become stronger, suggesting that crystalline cellulose partially degrades into amorphous cellulose. O’Connor et al. proposed the absorbance ratio of two bands, O’KI = A1430 cm^−1^/A893 cm^−1^, as the crystallinity index (CI) [54]. In order to eliminate the influence of baseline drift, the peak area rather than intensity is actually used in the CI formula. The calculated CI from FTIR spectra of the insulating paper decreases with increasing aging, as shown in Figure 10.

By comprehensively analyzing the changes in crystal structure and hydrogen bond strength of insulating paper, we can conclude that with the thermal aging process of insulating paper, the crystalline region is transformed into the amorphous region, the crystallinity index decreases, and the hydrogen bond strength of insulating paper increases. Through the analyses of Section 4.2 and Section 4.3, the reason for the increase in hydrogen bond strength was further explained.

### 4.2. Simulation of THz Spectra

Theoretically, the thermal aging process degrades crystalline cellulose, reduces its molecular density, and alters the adsorption location of water molecules along the molecular chain. All of these factors have an impact on the absorption characteristic. In this section, different densities of cellulose and three types of water–cellulose molecules were built to explain the change in the absorption behavior of cellulose molecules before and after the aging process. To comprehensively simulate the changes of cellulose before and after thermal aging, we considered the factors of density and moisture: firstly, the density was increased to analyze the process of crystalline transformed into amorphous cellulose qualitatively; secondly, three types of bound water were considered to describe the effects of moisture on the absorption characteristics.

**Figure 11:**
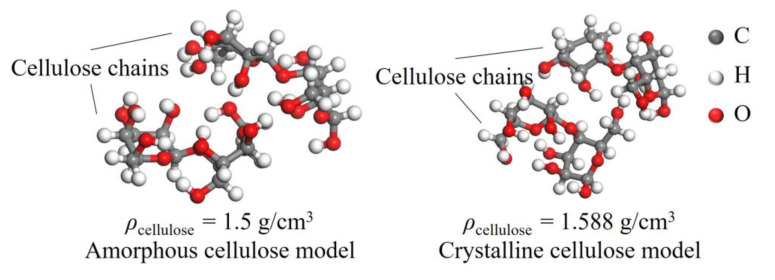
Cellulose molecular model with density of 1.5 g/cm^3^ (**left**) and 1.588 g/cm^3^ (**right**).

**Figure 12:**
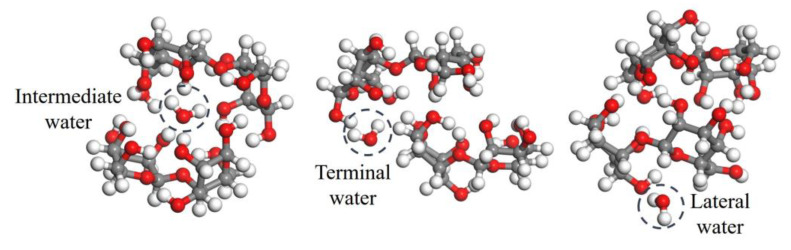
Three types of water–cellulose model with intermediate, terminal, and lateral water molecule.

The two sets of comparative models established are shown in Figure 11 and Figure 12 with cellobiose as the basic unit structure: (1) The average densities of cellulose amorphous region and crystalline region are 1.5 and 1.588 g/cm^3^, respectively, where the H-bond structure of cellulose molecules in the above two mentioned regions shows a big difference. Consequently 1.588 g/cm^3^ cellulose molecules represent the before-aging cellulose while 1.5 g/cm^3^ represents the after-aging cellulose. (2) The changes in the adsorption position of water molecules caused the changes in the H-bond structure, so three types of cellulose molecules with intermediate, terminal, and lateral water molecule are established. The following figures show the cellulose model, where the gray, white, and red sphere are carbon, hydrogen, and oxygen atom, respectively.

The theoretical absorption spectra of the molecular system above were calculated by the B3LYP method in density functional theory (DFT), and the 6-31G(d, p) basis set was selected for the geometric optimization and frequency calculation with the DFT-D3 dispersion correction, which can compensate for the shortcomings of B3LYP that cannot describe the weak interactions between molecules and improve the accuracy of the theoretical calculation. To make the calculation results more consistent with the experimental results, the correction factor of 0.952 fitted by Truhlar [55] was adopted in the analysis.

### 4.3. Comparison of Experimental and Theoretical Spectra

**Figure 13:**
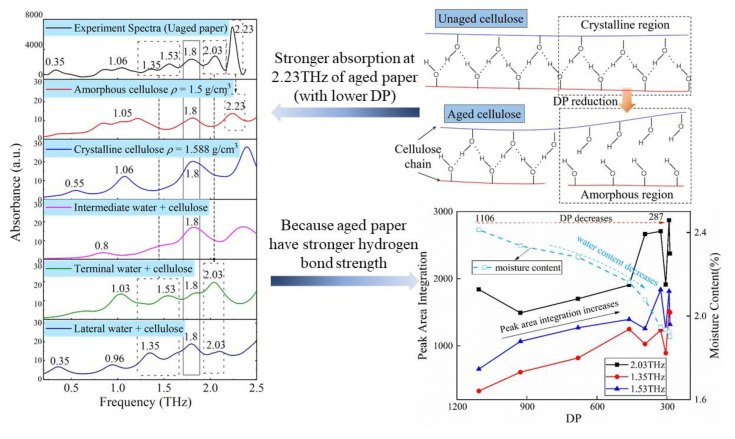
Correlation of DP reduction and terahertz absorption characteristics.

Figure 13 compares the measured spectrum with the simulated spectra of cellulose models. The absence of an imaginary frequency in the calculated spectra indicates that the optimized molecule conformation is stable. Over the frequency range of 0.2–2.5 THz, the vibrational modes of cellulose are primarily due to the wobbling, deformation, twisting, and rotational behavior of functional groups as opposed to stretching vibrations. This is because the bending of bond angles typically requires less activation energy than the change in bond length.

Over the range of 0.2 and 2.5 THz, the vibration mode of insulating paper is mainly the interaction between water and cellulose molecules. The absorption of insulating paper in the 0.8–1.2 THz band is due to the synergistic vibration of water and cellulose molecules. Further study of the experimental spectra revealed a limited association between the absorption of 0.8–1.2 THz and the DP value as shown in Figure 5b,c; hence, this paper does not discuss it in great detail. The absorption peaks at 1.35, 1.53, and 2.03 THz are one-on-one associated with the vibration of water–cellulose molecules having lateral, terminal, and intermediate water, respectively. FTIR analysis showed an increase in the crystalline region and H-bond strength of cellulose after thermal aging (seen in Figure 9), while THz absorption spectra showed an increase in the absorption peaks intensity associated with bound water (see lower right corner of Figure 13), i.e., 1.35, 1.53, and 2.03 THz. It indicates that the increase in the amorphous region leads to an increase in the H-bond strength of the bound water, which in turn enhances the overall H-bond strength of the insulating paper.

*S_1.8TH__z_* and *S_2.23TH__z_* are regarded as characteristic parameters to evaluate DP, and it is of great significance to further analyze the vibration modes of the two absorption peaks. All of the molecular models have an absorption peak at 1.8 THz, indicating that their vibration modes contain a range of complicated and synergistic water and cellulose vibrations. Section 3.2 also shows that the prediction accuracy of *S_1.8THz_* is lower than that of *S_2.23TH_*_z_, which is due to the influence of the vibration of bound water on the absorption of 1.8 THz. The FTIR analysis of insulating paper demonstrated that as the paper ages, crystalline cellulose decreases and amorphous cellulose increases, whereas the simulation spectrum of amorphous cellulose had a peak at 2.23 THz. Therefore, it is reasonable to attribute the vibration of amorphous cellulose to 2.23 THz. The thermal aging process demonstrates that the DP of cellulose molecules declines as the amorphous cellulose increases, which is also the reason why *S_2.23TH__z_* is a more precise parameter for predicting the DP value.

## 5. Conclusions

In this paper, the terahertz time-domain spectra of insulating paper with different DP are obtained, and the absorption characteristics of the samples are analyzed. Baseline correction and split-peak fitting algorithms are adopted to extract characteristic parameters for the quantitative evaluation of the DP value. Simulation and FTIR analysis are used to explain the vibration modes of absorption spectra. Therefore, the following conclusions can be drawn:

(1) After baseline correction and split-peak fitting algorithms, the absorption peaks of insulating paper at 1.8 THz and 2.23 THz show a positive correlation with thermal aging days. Through quantitative analysis *S_1.8THz_* and *S_2.23THz_* are both found to exist a log-linear relationship with DP value. The parameters’ universality is verified in two batches of thermal aging experiments with two types of insulating paper, the absolute error range of *S_1.8 THz_* and *S_2.23THz_* is 1.4–19.1% and 1.3–9.9%, respectively, which demonstrates that *S_2.23THz_* has higher accuracy in DP prediction.

(2) The FTIR of insulating paper aged 0 days, 3 days, and 128 days is obtained. It is demonstrated that thermal aging process increases the amorphous cellulose and hydrogen bond strength while decreasing the crystalline cellulose.

(3) In the analysis of vibration modes, two sets of cellulose models are first established to analyze the correlation of absorption characteristics with changes in water positions and cellulose structure. The density functional theory (DFT) of B3LYP/6-31G(d, p) basis set is selected to the geometric configurations and dynamics simulation. Calculation spectra show that the peak at 1.8 THz is assigned to the synergistic vibration of water and cellulose while the peak at 2.23 THz is assigned to amorphous cellulose. The decrease in DP of insulating paper leads to the increase in amorphous cellulose, which is the reason for the higher prediction accuracy of *S_2.23TH__z_*.

The method proposed in this paper has been verified in the insulating paper. The terahertz technique can reflect inter- and intra-molecular hydrogen bond oscillations; it is promising to extend this method into the properties detection of other cellulose/lignin composite materials, however, its applicability must be further examined.

## Data Availability

The data presented in this study are available on request from the corresponding author.
